# Hepatocellular carcinoma in hemodialysis patients

**DOI:** 10.18632/oncotarget.17127

**Published:** 2017-04-16

**Authors:** Chern-Horng Lee, Sen-Yung Hsieh, Chih-Chun Chang, I-Kuan Wang, Wen-Hung Huang, Cheng-Hao Weng, Ching-Wei Hsu, Tzung-Hai Yen

**Affiliations:** ^1^ Department of General Internal Medicine and Geriatrics, Chang Gung Memorial Hospital, Linkou, Taiwan; ^2^ Department of Gastroenterology and Hepatology, Chang Gung Memorial Hospital and College of Medicine, Chang Gung University, Linkou, Taiwan; ^3^ Department of Clinical Pathology, Far Eastern Memorial Hospital, Banciao, New Taipei City, Taiwan; ^4^ Department of Nephrology, China Medical University Hospital and College of Medicine, China Medical University, Taichung, Taiwan; ^5^ Department of Nephrology, Chang Gung Memorial Hospital and College of Medicine, Chang Gung University, Linkou, Taiwan; ^6^ Kidney Research Center, Chang Gung Memorial Hospital, Linkou, Taiwan; ^7^ Center for Tissue Engineering, Chang Gung Memorial Hospital, Linkou, Taiwan

**Keywords:** hepatocellular carcinoma, end-stage renal disease, hemodialysis, liver cirrhosis, tumor stage

## Abstract

We investigated the rates and predictors of mortality in hepatocellular carcinoma (HCC) patients who were or were not undergoing long-term hemodialysis. The participants in this retrospective observational study were 1298 HCC patients (60.0 ± 12.1 years old, 72% male), of whom 172 were undergoing hemodialysis and 1126 were not. HCC patients on hemodialysis exhibited a higher hepatitis C virus carrier rate (49.4% *versus* 39.3%, *P* = 0.012), lower hepatitis B virus carrier rate (37.2% *versus* 58.3%, *P* < 0.001) and lower hepatitis B or C virus carrier rate (77.9% *versus* 89.3%, *P* < 0.001) than those not on hemodialysis. Serum alkaline phosphatase levels were higher in the hemodialysis than non-hemodialysis group (162.8 ± 141.1 u/l *versus* 124.6 ± 102.5 u/l, *P* < 0.001). By the end of the analysis, 32.0% of HCC patients on hemodialysis and 28.0% of those not on hemodialysis had died. Kaplan-Meier analysis confirmed that cumulative survival was poorer in HCC patients on hemodialysis (*P* = 0.004). In a multivariate Cox regression model, hemodialysis (*P* < 0.001), older age (*P* < 0.001) and advanced tumor stages (*P* < 0.001) were found to be risk factors for mortality. HCC patients on hemodialysis had a 2.036-fold greater chance of death than HCC patients not on hemodialysis. Prospective studies with longer follow-ups and larger samples are warranted.

## INTRODUCTION

Renal disease is endemic in Taiwan; indeed, the incidence and prevalence of end-stage renal disease (ESRD) are greater in Taiwan than in any other country. In 2016, the Annual Data Report of the United States Renal Data System indicated that the incidence of treated ESRD remains the greatest in Taiwan, followed by the Jalisco region of Mexico and the United States (455, 421 and 370 cases per million population, respectively). In addition, the prevalence of treated ESRD was found to be the greatest in Taiwan, followed by Japan and the United States (3219, 2505, and 2076 cases per million population, respectively) [1].

Taiwan is also an endemic area for liver disease and HCC [2]. Hepatitis B virus and hepatitis C virus are the two most frequent etiologies of liver disease in Taiwan. The national prevalence rates among Taiwanese adults are 15-20% and 1-3% for hepatitis B and C viruses, respectively [3]. After a national neonatal vaccination program against hepatitis B was started in 1984, hepatitis B surface antigen became less prevalent among people under the age of 15, declining to 0.7% in 1999 from 9.8% in 1984. For people between the ages of 15 and 20, the hepatitis B surface antigen prevalence in 1999 was 7% [4].

In a review of dialysis registry data [5] from 10 regions in Asia and the Pacific (Australia, China, Hong Kong, India, Japan, Korea, Malaysia, New Zealand, Taiwan, and Thailand), it was noted that the prevalence rate of hepatitis C virus varied from 0.7 to 18.1% in different areas and was normally greater in hemodialysis than in peritoneal dialysis patients (7.9 ± 5.5% versus 3.0 ± 2.0%, *P* = 0.01). In addition, the hemodialysis population exhibited greater seroconversion rates than the peritoneal dialysis population (incidence rate ratio, peritoneal dialysis versus hemodialysis, 0.33; 95% confidence interval 0.13 – 0.75). On the other hand, hepatitis B virus infection is clearly less influenced by the dialysis modality [5].

Some studies [6–13] have evaluated mortality data from HCC patients in the ESRD population. However, the baseline characteristics of HCC patients with hemodialysis in Taiwan [14] may differ from those in other countries, where only a few patients are carriers of the hepatitis B virus. Although it is generally thought that survival is poorer in HCC patients with ESRD than in those without it [9, 11], some groups [6–8, 10, 12, 13] have reported that overall survival did not differ significantly between patients with and without dialysis. Therefore, we analyzed the rates and predictors of mortality in HCC patients who were or were not undergoing long-term hemodialysis.

## MATERIALS AND METHODS

### Ethics

This observational study conformed to the guidelines of the Declaration of Helsinki, and was approved by the Medical Ethics Committee of Chang Gung Memorial Hospital. Since this study involved the retrospective review of existing data, Institutional Review Board approval was obtained without specific informed consent from the patients. In addition, all individual data were securely protected through the delinking of identifying information from the main data set, and were only available to the investigators. All data were analyzed anonymously. The Institutional Review Board of the Chang Gung Memorial Hospital specifically waived the need for consent. The study approach was based on previous studies [15, 16].

### Patients

In total, 1298 HCC patients seen at Chang Gung Memorial Hospital between 2000 and 2007 were enrolled and participated in this single-center cohort study. The subjects were categorized into two subgroups based on whether they were (*N* = 172) or were not (*N* = 1126) undergoing hemodialysis. Typically, patients were followed at 2-month intervals for the first 2 years and then every 3-6 months thereafter. Patients’ baseline characteristics and clinical and laboratory data were obtained for analysis, and mortality rates were analyzed.

### Inclusion and exclusion criteria

All HCC patients 18 years old or older were enrolled in this study. Patients were excluded from this study if they had chronic kidney disease (stage 3, 4 or 5 [non-dialysis]) [2], other malignancies [17], lead [18] or cadmium [19] intoxication, had undergone renal transplantation in the previous 3 months, had undergone hemodialysis for less than 3 months, or had HCC before hemodialysis. As the International Agency for Research on Cancer lists lead and cadmium as Group 2B and 1 carcinogens, respectively [20], patients with lead and cadmium intoxication were excluded from this study.

### Diagnosis of chronic kidney disease

The estimated glomerular filtration rate was calculated with the 4-variable chronic kidney disease epidemiology collaboration equation [21]. The chronic kidney disease stages were defined as 1, 2, 3, 4 and 5 according to estimated glomerular filtration rates > 90, 60 - 90, 30 - 60, 15 - 30, and < 15 ml/min per 1.73 m^2^, respectively.

### Hemodialysis sessions

All ESRD patients underwent 4 hours of hemodialysis, three times per week. Hemodialysis was performed with single-use hollow-fiber dialyzers equipped with modified cellulose-based polyamide or polysulfone membranes. The dialysate used for all patients had a standard ionic composition and was a bicarbonate-based buffer.

### Diagnosis of HCC

At our hospital, HCC was clinically diagnosed by various imaging modalities, including ultrasonography, radiocontrast enhanced triphasic dynamic computed tomography, magnetic resonance imaging, angiography, and/or documented tissue histopathology [22]. A liver tumor biopsy was not required as long as the typical clinical and imaging features of HCC were present.

### Diagnosis of liver cirrhosis

Cirrhosis was diagnosed by pathology or by laboratory tests, liver ultrasonography, and clinical features of chronic hepatitis with portal hypertension (varices, thrombocytopenia, splenomegaly) and/or liver decompensation (jaundice, prolonged prothrombin time, ascites) [23].

### Barcelona Clinic Liver Cancer (BCLC) staging system

BCLC stage 0 encompasses patients with Child-Pugh A liver disease diagnosed with one nodule measuring < 2 cm, without vascular invasion or satellites. BCLC stage A includes patients with Child-Pugh A or B disease diagnosed with one nodule of any size or a maximum of three nodules measuring < 3 cm. BCLC stage B comprises patients with Child-Pugh A or B disease diagnosed with multiple nodules, without vascular invasion or extrahepatic metastasis. Patients with Child-Pugh A or B disease, vascular invasion or extrahepatic metastasis and cancer-related symptoms (performance status test 1-2) are classified as having BCLC stage C disease. Patients with Child-Pugh C disease in any tumor stage and with cancer-related symptoms (performance status test > 2) are grouped as having BCLC stage D disease [24].

### Management of HCC

At our hospital, HCC patients with BCLC stage 0 and A disease underwent tumor resection surgery, liver transplantation, or interventional therapies for local cancer ablation, including radiofrequency ablation, ethanol injection therapy, acetic acid injection therapy and transcatheter arterial chemo-embolization [25]. Patients with BCLC stage B disease underwent transcatheter arterial chemo-embolization and radiofrequency ablation [25]. Patients with BCLC stage C disease received palliative chemotherapy, transcatheter arterial chemo-embolization or radiotherapy, along with supportive medical care, and those with BCLC stage D disease received symptomatic palliative care [25].

### Statistical analysis

Continuous variables are expressed as means and standard deviations, while categorical variables are expressed as numbers with percentages in brackets. For comparisons between patient groups, Student's t test was used for continuous variables, and chi-square or Fisher's exact tests were used for categorical variables. Mortality data were examined with the Kaplan-Meier method, and the significance was assessed with a log-rank test. Univariate Cox regression analysis was performed to compare the frequency of risk factors associated with mortality. To avoid confounding factors, we performed multivariate Cox regression analysis to assess the factors that were significant in the univariate models and met the assumptions of a proportional hazards model. We considered results that rejected the null hypothesis with 95% confidence to be significant. All analyses were performed with IBM SPSS Statistics Version 20.

## RESULTS

The HCC patients were 60.0 ± 12.1 years old and mostly male. Not all patients underwent liver tumor biopsies. Histopathological proof was available for 71 (41.3%) of the 172 HCC patients with hemodialysis and 352 (31.3%) of the 1126 patients without hemodialysis. Table 1 demonstrates that the hepatitis B virus, hepatitis C virus, and hepatitis B or C virus prevalence rates were high. HCC patients on hemodialysis had a higher hepatitis C virus carrier rate (49.4% versus 39.3%, *P* = 0.012), lower hepatitis B virus carrier rate (37.2% versus 58.3%, *P* < 0.001) and lower hepatitis B or C virus carrier rate (77.9% versus 89.3%, *P* < 0.001) than HCC patients without hemodialysis. The serum alkaline phosphatase level was higher in the hemodialysis group than in the non-hemodialysis group (162.8 ± 141.1 u/l versus 124.6 ± 102.5 u/l, *P* < 0.001).

**Table 1:** Baseline characteristics and laboratory data of HCC patients (*N*=1298)

Variable	HCC with hemodialysis (*N*= 172)	HCC without hemodialysis (*N*= 1126)	*P* value
Age, years	60.0±12.3	59.8±11.1	0.806
Male, *n* (%)	114 (66.3)	820 (72.8)	0.075
Hepatitis B virus carrier, *n* (%)	64 (37.2)	656 (58.3)	<0.001***
Hepatitis C virus carrier, *n* (%)	85 (49.4)	443 (39.3)	0.012*
Hepatitis B or C virus carrier, *n* (%)	134 (77.9)	1006 (89.3)	<0.001***
Hepatitis B and C virus carrier, *n* (%)	15 (8.7)	99 (8.8)	0.975
Creatinine, mg/dL	8.4±2.7	0.9±0.2	0.000***
Alpha fetoprotein, ng/ml	6650.4±40815.8	13755.2±143461.9	0.560
T-bilirubin, mg/dl	1.1±2.1	1.7±2.7	0.009**
Alkaline phosphatase, U/L	162.8±141.1	124.6±102.5	0.000***
Albumin, g/dl	3.4±0.7	3.7±3.2	0.338
Prolonged prothrombin time, sec	1.7±3.6	1.8±3.0	0.644
Platelet count, x10^3^/μL	138.8±85.8	148.8±91.3	0.083

Note: *P < 0.05, **P < 0.01, ***P < 0.001

As shown in Tables 2 and 3, there were no significant differences in the Child-Pugh liver cirrhosis scores (*P* = 0.280) and BCLC tumor stages (*P* = 0.056) between HCC patients with and without hemodialysis.

**Table 2:** Liver cirrhosis classification of HCC patients (*N* = 1298)

Variable	HCC with hemodialysis (*N*= 172)	HCC without hemodialysis (*N*= 1126)	*P* value
0, *n* (%)	45 (26.2)	227 (20.2)	0.280
Class A, *n* (%)	82 (47.7)	603 (53.6)	
Class B, *n* (%)	35 (20.3)	219 (19.4)	
Class C, *n* (%)	10 (5.8)	77 (6.8)	

Note: The liver cirrhosis classification was based on the Child-Pugh score.

**Table 3:** Tumor staging of HCC patients (*N* = 1298)

Variable	HCC with hemodialysis (*N*=172)	HCC without hemodialysis (*N*=1126)	*P* value
Stage 0	16 (9.3)	132 (11.7)	0.056
Stage A	70 (40.7)	344 (30.6)	
Stage B	54 (31.4)	361 (32.1)	
Stage C	21 (12.2)	211 (18.7)	
Stage D	11 (6.4)	78 (6.9)	

Note: Tumor staging was based on the Barcelona Clinic Liver Cancer staging system.

**Figure 1:**
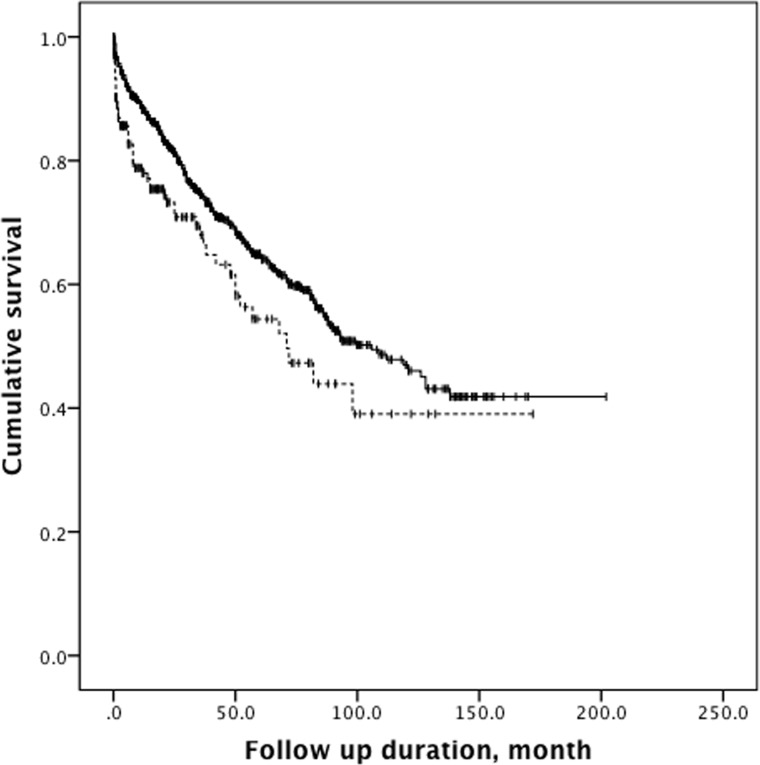
Kaplan-Meier analysis The analysis demonstrates that HCC patients with hemodialysis (dashed line) had significantly poorer cumulative survival than HCC patients without hemodialysis (solid line) (log-rank test, chi-square 8.152, *P* = 0.004).

HCC patients with hemodialysis were followed for 30.0 ± 32.5 months, whereas those without hemodialysis were followed for 37.6 ± 37.4 months (*P* < 0.001, Table 4). By the end of the analysis, 55 (32.0%) of the 172 HCC patients with hemodialysis and 314 (28.0%) of the 1126 HCC patients without hemodialysis had died. The mortality rates of the groups did not differ significantly in chi-square analysis (*P* = 0.287). Nevertheless, Kaplan-Meier survival analysis revealed that cumulative survival was significantly poorer in HCC patients with hemodialysis than in HCC patients without hemodialysis (log-rank test, chi-square = 8.152, *P* = 0.004, Figure 1). The 1-, 3-, and 5-year survival rates were 78.0%, 67.9% and 54.4% for HCC patients with hemodialysis and 88.3%, 74.5% and 64.8% for those without hemodialysis.

**Table 4:** Outcomes of HCC patients (*N* = 1298)

Variable	HCC with hemodialysis (*N*= 172)	HCC without hemodialysis (*N*= 1126)	*P* value
Follow-up duration, months	30.0±32.5	37.6±37.4	<0.001***
Mortality, *n* (%)	55 (32.0)	314 (28.0)	0.287

Note: ***P < 0.001.

In a multivariate Cox regression model (Table 5), hemodialysis (odds ratio [OR] 2.036, 95% confidence interval [CI], 1.515 - 2.740, *P* < 0.001), older age (OR 1.019, 95% CI 1.010 - 1.029, *P* < 0.001) and advanced BCLC tumor stages (*P* < 0.001) were identified as significant risk factors for mortality. HCC patients with hemodialysis had 2.036-fold higher odds of death than HCC patients without hemodialysis.

**Table 5:** Cox regression analysis of mortality (*N* = 1298)

Variable	Multivariate analysis	
	Odds ratio (95% confidence interval)	*P* value
Hemodialysis (yes)	2.036 (1.515 - 2.740)	<0.001***
Age (each increase of one year)	1.019 (1.010 - 1.029)	<0.001***
Tumor staging, Stage 0 (as reference)		<0.001***
Stage A	0.813 (0.517 - 1.279)	0.370
Stage B	0.801 (0.533 - 1.202)	0.283
Stage C	1.500 (1.002 - 2.246)	0.049*
Stage D	4.444 (2.977 - 6.634)	<0.001***

Note: *P < 0.05, ***P < 0.001

## DISCUSSION

The major finding of this large cohort study of 1298 HCC patients at a single center was that hemodialysis (*P* < 0.001), older age (*P* < 0.001) and advanced tumor stages (*P* < 0.001) significantly increased the odds of mortality. Since the prognosis of patients with HCC generally depends on patient-related variables (such as age or comorbidity) and tumor-related variables (such as tumor stage) [26], it is easy to understand why these three factors were independent predictors of mortality in our patients.

As mentioned, some studies [6–13] have evaluated the mortality data of HCC patients in ESRD populations (Table 6). In one study [6], clinical data and operative results were compared between 12 HCC patients with ESRD and 456 patients without ESRD. The two groups were similar in terms of operative morbidity and mortality. The 5-year disease-free survival rate was 35.0% for the ESRD group and 34.2% for the non-ESRD group (*P* = 0.31), and the 5-year actuarial survival rate was 67.8% for the ESRD group and 53.3% for the non-ESRD group (*P* = 0.54) [6]. Tung et al. [7] analyzed 13 patients who were receiving hemodialysis and had been diagnosed with HCC, and found that six of them had hepatitis B virus, while seven had hepatitis C virus. There were no significant differences in the serum alanine aminotransferase, alpha-fetoprotein, aspartate aminotransferase and bilirubin levels in patients with HCC caused by hepatitis B virus or hepatitis C virus. Furthermore, the survival curves did not differ significantly between the groups receiving treatment (hepatic resection and/or transcatheter arterial chemoembolization) or supportive treatment (*P* = 0.69) [7].

**Table 6:** Published studies of HCC patients undergoing long-term hemodialysis

Study	Year	Geographic area	Sample size	Hemodialysis patients	Source of data	Survival disadvantage of hemodialysis
Cheng et al. [6]	2001	Taiwan	468	12	Hospital records	No
Tung et al. [7]	2003	Taiwan	13	13	Hospital records	No
Yeh et al. [8]	2005	Taiwan	1224	26	Hospital records	No
Orii et al. [9]	2008	Japan	68	15	Hospital records	Yes
Kondo et al. [10]	2009	Japan	14	14	Hospital records	
Hwang et al. [11]	2012	Taiwan	77428	38714	National Health Insurance	Yes
Yeh et al. [12]	2013	Taiwan	5746	149	National Health Insurance	No
Lee et al. [13]	2013	Taiwan	2502	30	Hospital records	No
Present study	2016	Taiwan	1298	172	Hospital records	Yes

Similarly, Yeh et al. [8] reported that the overall and disease-free survival rates were similar between 26 HCC patients with ESRD and 1198 HCC patients without ESRD, all of whom had undergone hepatic resection during the same interval [8]. In another study, Orii et al. [9] reviewed the records of 17 chronic kidney disease patients (15 with ESRD) and 51 non-chronic kidney disease patients who all had undergone hepatectomy for HCC during the same timeframe. Postoperative circulatory problems developed more often in the chronic kidney disease group (*P* = 0.013). While the groups exhibited similar disease-free survival rates, the chronic kidney disease had a lower overall survival rate than the non-chronic kidney disease group (*P* = 0.031) [9]. Kondo et al. [10] assessed outcomes after radiofrequency ablation of HCC in patients with ESRD undergoing regular hemodialysis. Intraperitoneal hemorrhaging and other such complications were not observed. After a single treatment, local tumor progression was noted, but successful management was achieved by repeat radiofrequency ablation. Over a 343-day mean observation period, only one of the five patients with naive tumors died (of heart failure) [10].

In another population-based study [11], all patients with ESRD who received dialysis between 2003 and 2007 (*n* = 38714) were enrolled. Controls (*n* = 38714) matched for age, gender, hepatitis B and C infection, and liver cirrhosis during the same period were chosen from a database of 1 million randomly selected subjects. The incidence of HCC did not differ significantly between the patients with ESRD and the non-ESRD controls (2.03 per 1000 person-years versus 2.10 per 1000 person-years, rate ratio = 0.947; 95% confidence interval: 0.792–1.132). A higher proportion of ESRD patients than non-ESRD controls had diabetes mellitus, gout, heart failure and hypertension (*P* < 0.001). The cumulative survival rate of those with HCC was also worse in the ESRD than in the non-ESRD group (*P* < 0.001). ESRD patients developing HCC were younger and had more comorbidities than their non-ESRD counterparts. Diabetes mellitus (hazard ratio = 1.55) and ESRD (hazard ratio = 1.61) were predictive of mortality in these HCC patients [11].

Yeh et al. [12] compared the survival, perioperative mortality and complications of 149 HCC patients with ESRD and 596 HCC patients without ESRD who received hepatic surgery. Among the HCC patients with ESRD, the 1-, 5-, and 10-year overall survival rates were 86, 52, and 38%, and the disease-free survival rates were 77, 27, and 18%, respectively. The HCC cohorts with and without ESRD exhibited similar survival rates. The risk of postoperative infections requiring intervention and the risk of heart problems were both greater in the HCC cohort with ESRD than in the cohort without ESRD [12].

Finally, in a study of 2502 HCC patients [13], data were reviewed from 30 dialysis patients and 90 controls matched for age, sex and treatment. Dialysis patients exhibited higher rates of dual viral hepatitis B and C, lower serum alpha-fetoprotein levels, poorer performance status and greater model for end-stage liver disease scores than non-dialysis patients or matched controls (*P* < 0.05). Survival did not differ significantly between dialysis and non-dialysis patients (*P* = 0.684) or between dialysis patients and matched controls (*P* = 0.373). A Cox regression model revealed that dialysis for < 40 months (*P* = 0.019) and ascites (*P* = 0.019) independently predicted poor prognosis among dialysis patients who had HCC [13].

In this study, the HCC patients on hemodialysis had a higher hepatitis C virus carrier rate (49.4% versus 39.3%, *P* = 0.012), lower hepatitis B virus carrier rate (37.2% versus 58.3%, *P* < 0.001) and lower hepatitis B or C virus carrier rate (77.9% versus 89.3%, *P* < 0.001) than HCC patients without hemodialysis. Hepatitis B and hepatitis C are two pathogenic viruses that frequently cause chronic hepatitis in ESRD patients. As screening programs for hepatitis B surface antigen have become common over the past 30 years, carrier patients have been identified and isolated. Thus, hepatitis B is seen less often in dialysis units at present [14]. Hepatitis C virus antibody positivity is associated with a history of blood transfusions and with the therapeutic mode and duration of hemodialysis [27]. In Taiwan, the annual seroconversion rate of the hepatitis C virus antibody in dialysis patients has been reported to be as high as 15.0% [14]. Whether patients infected with hepatitis C virus should be identified and isolated during hemodialysis treatment is an issue of debate. This might explain the higher hepatitis C virus antibody positivity in HCC patients undergoing hemodialysis.

Nevertheless, there is no clear explanation for the lower hepatitis B or C virus carrier rate in HCC patients with hemodialysis, although it may be related to the immune status or toxin accumulation of uremic patients. Theoretically, the risk factors for HCC formation include hepatitis B or C virus infections, alcoholic liver disease, nonalcoholic fatty liver disease, hereditary hemochromatosis, alpha 1-antitrypsin deficiency, autoimmune hepatitis, certain porphyrias and Wilson's disease [28]. ESRD is concurrently associated with immune activation and immune deficiency [29], and the immune dysfunction in uremia is always linked with alterations in innate and adaptive immunity [30]. While these immune disorders are complicated and not fully understood, progressive defects of the immune system promote both side effects and causes of mortality such as infections, cardiovascular problems, and possibly malignancy [31].

In addition, reduced renal function and the ensuing uremia increase the plasma concentrations of uremic toxins, which more frequent dialysis treatment can reduce but not completely remove [31, 32]. The European Uremic Toxin Work Group has listed toxins/substances thought or known to exhibit biological activity that accumulate in patients with ESRD [33]. These uremic toxins/substances have been classified into three groups based on their molecular weights, capacity to bind proteins, and pattern of removal by dialysis. However, their potential toxicity and carcinogenicity remain to be elucidated [34]. For example, in ESRD patients with immune deficiency, T-cell lymphopenia and a decline in plasmacytoid dendritic cells and natural killer cells could trigger insufficient responses to viruses and reduced tumor surveillance. Reduced surveillance has important clinical implications because these patients are at greater risk for tumors thought or known to result from viral infections [35–37].

In this study, the serum alkaline phosphatase level was higher in HCC patients with hemodialysis than in those without hemodialysis (162.8 ± 141.1 u/l versus 124.6 ± 102.5 u/l, *P* < 0.001). Liver, bone, kidney, intestine and placenta all have high alkaline phosphatase levels [38]. The serum alkaline phosphatase in adults mainly originates in the liver and bone. The circulating concentration of alkaline phosphatase rises during pregnancy due to increases in the placental levels of this compound, and may double or even quadruple by the third trimester. Therefore, for HCC patients with hemodialysis, alkaline phosphatase is simply a biochemical marker of bone turnover, and its elevation usually indicates high-turnover bone disease or osteitis fibrosa cystica resulting from secondary hyperparathyroidism [39]. The association of high serum alkaline phosphatase levels with increased all-cause and cardiovascular mortality is well-established in the hemodialysis population [40].

In conclusion, HCC patients with hemodialysis had 2.036-fold higher odds of death than HCC patients without hemodialysis. The limitations of this study include its retrospective nature, short follow-up duration, small sample size, lack of parathyroid hormone blood testing, and most importantly, lack of a hemodialysis control group. Thus, further studies that address these limitations are warranted.
